# Phase separation of α-crystallin-GFP protein and its implication in cataract disease

**DOI:** 10.1038/s41598-023-31845-9

**Published:** 2023-03-24

**Authors:** Jie Shi, Ya-Xi Zhu, Rui-Yan Huang, Shao-Mei Bai, Yu-Xing Zheng, Jian Zheng, Zhao-Xia Xia, Yun-Long Wang

**Affiliations:** 1 Department of Radiation Oncology, The Sixth Affiliated Hospital, Sun Yat-sen University, Guangzhou, 510655 Guangdong China; 2 Guangdong Provincial Key Laboratory of Colorectal and Pelvic Floor Diseases, The Sixth Affiliated Hospital, Sun Yat-sen University, Guangzhou, 510655 Guangdong China; 3 Department of Pathology, The Sixth Affiliated Hospital, Sun Yat-sen University, Guangzhou, 510655 Guangdong China; 4 Guangdong Institute of Gastroenterology, Guangzhou, 510655 Guangdong China; 5 Department of Ophthalmology, The Sixth Affiliated Hospital, Sun Yat-sen University, Guangzhou, 510655 Guangdong China; 6 Department of Radiation Oncology, The Sixth Affiliated Hospital, Sun Yat-sen University, Guangzhou, 510655 Guangdong China

**Keywords:** Eye diseases, Biophysics, Cell biology

## Abstract

Cataract, the leading cause of blindness worldwide, is caused by crystallin protein aggregation within the protected lens environment. Phase separation has been implicated as an important mechanism of protein aggregation diseases, such as neurodegeneration. Similarly, cataract has been proposed to be a protein condensation disease in the last century. However, whether crystallin proteins aggregate via a phase separation mechanism and which crystallin protein initiates the aggregation remain unclear. Here, we showed that all types of crystallin-GFP proteins remain soluble under physiological conditions, including protein concentrations, ion strength, and crowding environments. However, in age or disease-induced aberrant conditions, α-crystallin-GFP, including αA- and αB-crystallin-GFP, but not other crystallin-GFP proteins, undergo phase separation in vivo and in vitro. We found that aging-related changes, including higher crystallin concentrations, increased Na^+^, and decreased K^+^ concentrations, induced the aggregation of α-crystallin-GFP. Furthermore, H_2_O_2_, glucose, and sorbitol, the well-known risk factors for cataract, significantly enhanced the aggregation of αB-crystallin-GFP. Taken together, our results revealed that α-crystallin-GFP forms aggregates via a phase transition process, which may play roles in cataract disease. Opposite to the previously reported function of enhancing the solubility of other crystallin, α-crystallin may be the major aggregated crystallin in the lens of cataract patients.

## Introduction

Cataract is the leading cause of blindness, which accounts for one third of visual impairments and about half of blindness cases worldwide^1^. Cataract is caused by the aggregation of crystallin proteins in the eye lens. The lens is a nearly transparent biconvex structure that is suspended behind the iris of each eye and it is partially responsible for the focusing of light onto the retina. Therefore, the insoluble protein aggregates in the lens block the light and impair visibility. Crystallin proteins consists of the members of the α-, β-, and γ-crystallin families, and they account for 90% of the proteins in the mature lens^2^. α-crystallin is a member of the small heat shock protein family and it serves as an ATP-independent chaperone that efficiently binds to damaged or partially unfolded proteins to prevent widespread protein aggregation. β- and γ-crystallin have a common two-domain structure comprising four repeated “Greek key” motifs, which are essential for their high stability^3^. All of these types of crystallin proteins have been found in the aggregates of cataract patients. Although several risk factors for cataract have been identified, including oxidation environments, diabetes, and radiation exposure^4^, the detailed mechanism underlying crystallin protein aggregation remains poorly understood.

In the last decade, phase separation has been implicated as an important mechanism of protein aggregation in neurodegenerative disease, such as Parkinson’s and Alzheimer’s diseases^5^. For example, FUS, a protein with a prion-like domain at its N-terminal, undergoes liquid–liquid phase separation to form highly concentrated droplets or condensates in cells. This condensate has liquid properties and can be resolved again. However, when age increases, the liquid-like FUS condensates will develop a solid-like phase or aggregates, which are hard to be resolved^6^. Similar to neurodegenerative diseases, cataract has been proposed to be a protein condensation disease in the last century, due to observations that crystallin proteins could separate into a protein-rich region and protein-poor region^7^. However, whether crystallin proteins aggregate via a phase separation mechanism remains unclear.

Here, our data indicate that α-crystallin-GFP proteins form aggregates via a phase separation mechanism in vitro and in vivo. Specifically, both αA- and αB-crystallin-GFP proteins form aggregates under the condition of aging-related cataracts. Whereas, under the conditions of oxidation- or diabetes-induced cataracts, αB-crystallin-GFP is the major aggregated crystallin.

## Results

### α-Crystallin-GFP forms puncta in SRA01/04 and HLE-B3 cells

**Figure 1:**
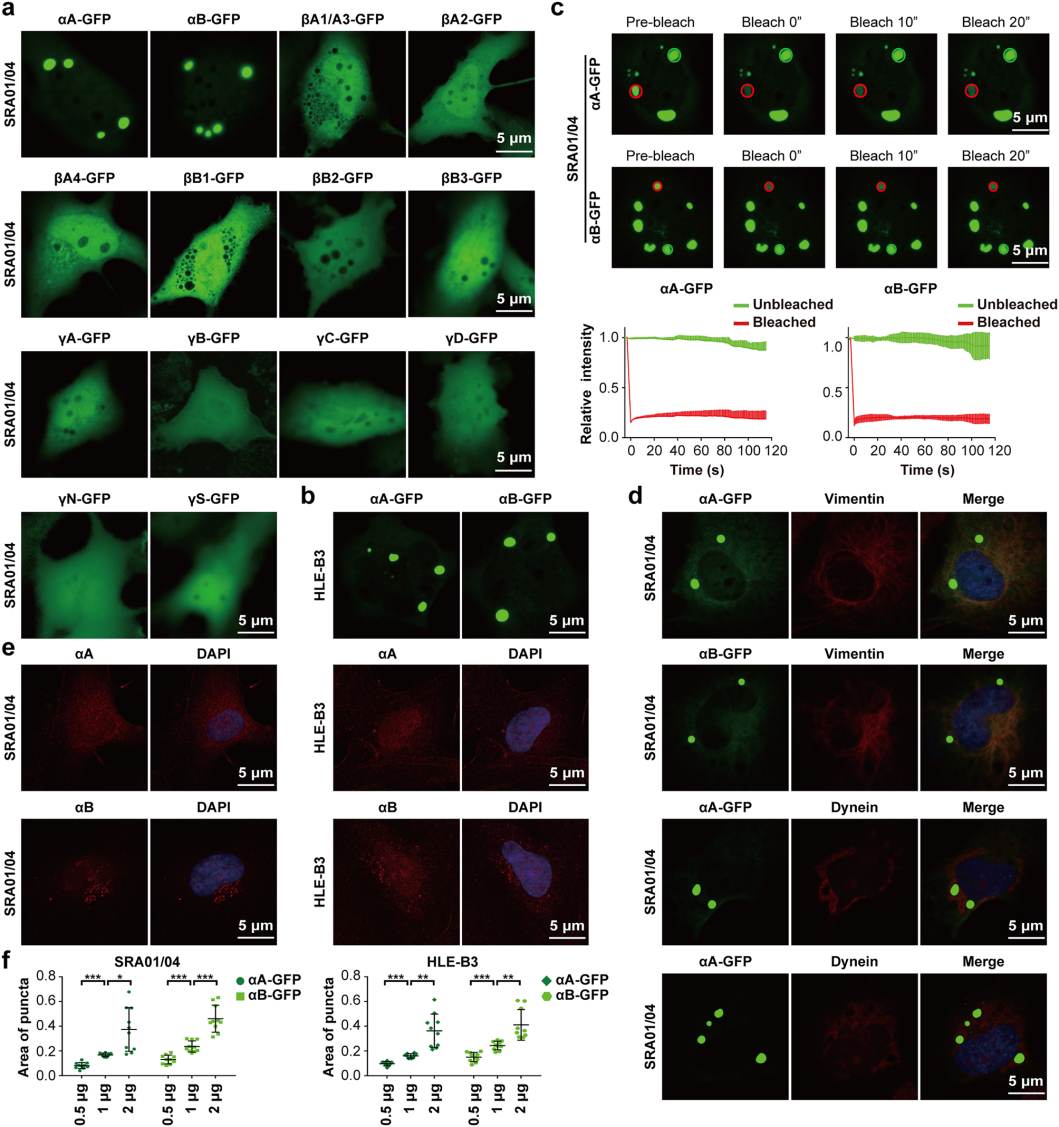
α-Crystallin-GFP form puncta in lens epithelial cells. (**a**,**b**) Ectopic expression of crystallin-GFP recombinant proteins in SRA01/04 and HLE-B3 cells. (**c**) FRAP assay showed that αA- and αB-crystallin-GFP condensates contained no mobile fraction in SRA01/04 cells. The bleached punctum was labeled by red circle and unbleached control punctum were labeled by green circle. Data are mean ± SD from 3 independent puncta. Three bleached puncta were included. (**d**) αA- and αB-crystallin-GFP puncta were not co-localized with vimentin and dynein in SRA01/04 cells. For (**a**–**d**), cells were transfected with 1 μg plasmids. (**e**) Immunofluorescence of endogenous αA- and αB-crystallin in SRA01/04 and HLE-B3 cells. (**f**) Overexpression of αA- and αB-crystallin-GFP recombinant proteins in different amounts of plasmid (0.5 μg, 1 μg and 2 μg) in SRA01/04 and HLE-B3 cells. Data are the mean ± SD. n = 10 images for each group. **P < 0.01; ***P < 0.001.

To examine whether crystallin proteins underwent phase separation, members of the crystallin-GFP proteins were ectopically expressed in SRA01/04 cells, an immortalized human lens epithelial cell line. Interestingly, only αA- and αB-crystallin-GFP formed high concentrated puncta, whereas other crystallin-GFP remained diffuse in cells (Fig. 1a). The formation of αA- and αB-crystallin-GFP puncta was further verified in another human lens epithelial cell line HLE-B3 (Fig. 1b). A fluorescence recovery after photobleaching (FRAP) assay was performed to examine the dynamic exchange between puncta and diffused phase. αA- and αB-crystallin-GFP puncta were photobleached and continuously observed for 120 s. Surprisingly, almost no fluorescence recovery of αA- or αB-crystallin-GFP was found after photobleaching (Fig. 1c), suggesting that those condensates were solid-like phases or aggregates. We next asked whether α-crystallin-GFP condensates exhibited features of aggresomes, the pericentriolar accumulations of aggregated protein^8^. As shown in Fig. 1d, α-crystallin puncta did not co-localize with vimentin and dynein, two known components of aggresomes, suggesting that α-crystallin-GFP condensates were not aggresomes. Surprisingly, immunofluorescence assays showed that endogenous αA- and αB-crystallin did not form puncta in SRA01/04 and HLE-B3 cells (Fig. 1e). We thought the different phenotypes between endogenously and exogenously expressed αA- and αB-crystallin may be ascribed to the lower level of endogenous αA- and αB-crystallin. Consistently, reducing the amount of plasmid significantly decreased the puncta formation of exogenously expressed αA- and αB-crystallin-GFP (Fig. 1f).

### Crystallin-GFP remains soluble in physiological conditions in vitro

**Figure 2:**
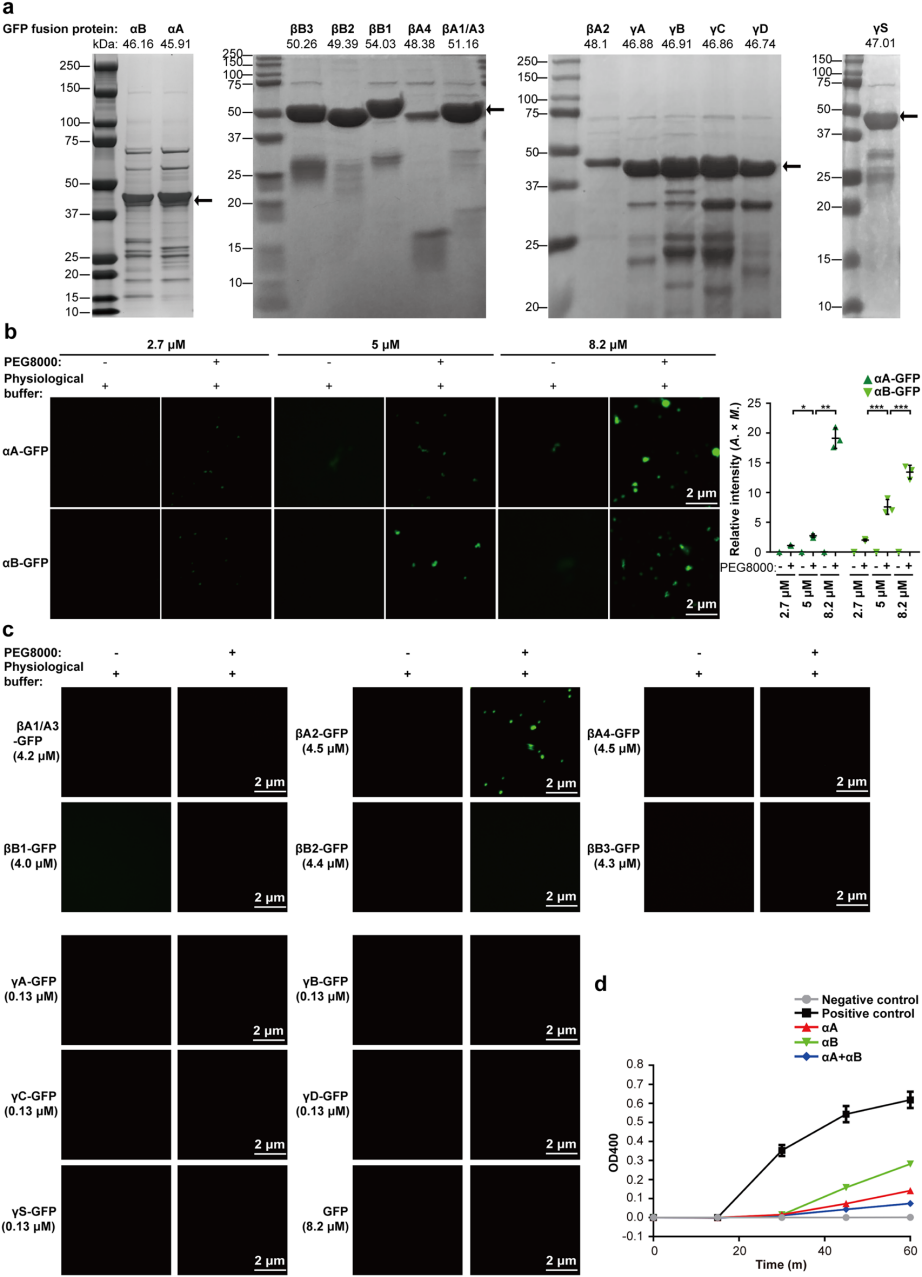
In vitro aggregation of recombinant crystallin-GFP proteins in physiological buffer. (**a**) The purified crystallin-GFP recombinant proteins were verified with Coomassie Staining, including αA-, αB-, βA1/A3-, βA2-, βA4-, βB1-, βB2-, βB3-, γA-, γB-, γC-, γD-, and γS-crystallin-GFP proteins. (**b**) The liquid-to-solid phase transition of αA- and αB-crystallin-GFP proteins (2.7 μM, 5 μM, or 8.2 μM) were explored in physiological buffer (20 mM NaCl, 10 μM CaCl_2_, 20 mM KCl, 20 mM Tris–HCl pH7.4, 0.6 mM glucose, 12 mM glutathione, 1 mM vitamin C, and 5.9 mM inositol), and with or without 5% PEG8000. The fluorescence intensity is presented as the area × mean intensity (*A.* × *M.*). Data are the mean ± SD. n = 3 images for each group. **P < 0.01. (**c**) The aggregation of βA1/A3 (4.2 μM)-, βA2 (4.5 μM)-, βA4 (4.5 μM)-, βB1 (4.0 μM)-, βB2 (4.4 μM)-, βB3 (4.3 μM)-, γA (0.13 μM)-, γB (0.13 μM)-, γC (0.13 μM)-, γD (0.13 μM)-, and γS (0.13 μM)-crystallin-GFP recombinant proteins in vitro was examined in physiological buffer, with or without 5% PEG8000. For (**b**,**c**), the incubation time was 10 min at 4 °C. (**d**) An insulin aggregation assay of chaperone activity of purified recombinant αA- or αB-crystallin-GFP proteins at 25 °C. Assay mixture (200 μl) contained insulin (0.35 mg/mL), αA-crystallin-GFP (1.05 mg/mL), or αB-crystallin-GFP (0.35 mg/mL) or both of αA- and αB-crystallin-GFP, and 20 mM DTT in 50 mM PBS (pH 7.2) were monitored after the indicated incubation time (0, 15, 30, 45, or 60 min).

To verify the aggregation of crystallin proteins in vitro, we expressed and purified most types of crystallin-GFP recombinant proteins from *E. coli*, including αA-, αB-, βA1/A3-, βA2-, βA4-, βB1-, βB2-, βB3-, γA-, γB-, γC-, γD-, and γS-crystallin-GFP proteins (Fig. 2a). It has been reported that in normal lenses, crystallin proteins were dissolved in a buffer containing 20 mM Na^+^, 10 μM Ca^2+^, and 120 mM K^+^ (physiological buffer)^9,10^. The purified recombinant proteins were diluted in a physiological buffer to a final concentration of 2.7 μM (αA/B), 4.0–4.5 μM (βA/B), and 0.13 μM (γA/B/C/D/S). The concentration of these purified crystallin-GFP proteins were calculated according to the mass percentage of crystallin, which accounted for ∼90% of the ocular proteins in the lens^2^. In agreement with the high solubility of crystallin, all crystallin-GFP proteins remained soluble and no aggregates formed (Fig. 2b,c). To verify the quality of purified proteins, the chaperone activity of purified αA- and αB-crystallin-GFP was examined. As shown in Fig. 2d, both αA- and αB-crystallin-GFP prevented the DTT-induced aggregation of insulin, indicating good quality purified proteins.

### α-Crystallin-GFP is the major aggregated crystallin of aging-related cataracts

**Figure 3:**
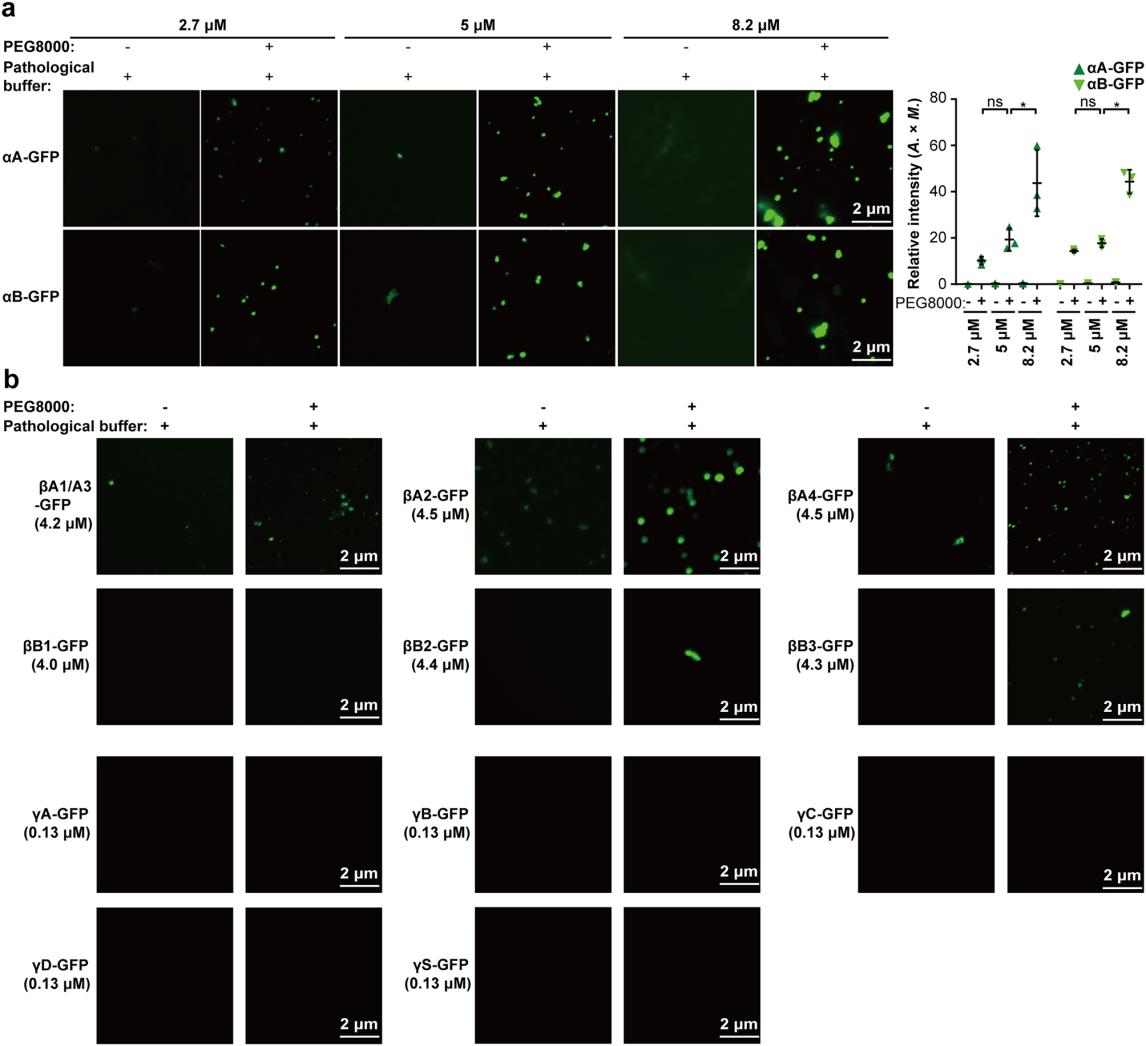
In vitro aggregation of recombinant crystallin-GFP proteins in pathological buffer. (**a**) The liquid-to-solid phase transition of αA- and αB-crystallin-GFP proteins (2.7 μM, 5 μM, or 8.2 μM) were detected in pathological buffer (150 mM NaCl, 30 mM CaCl_2_, 5 mM KCl, 20 mM Tris–HCl pH7.4, 0.6 mM glucose, 12 mM glutathione, 1 mM vitamin C, and 5.9 mM inositol), and with or without 5% PEG8000. The fluorescence intensity is presented as the area × mean intensity (*A.* × *M.*). Data are the mean ± SD. n = 3 images for each group. (**b**) The aggregation of βA1/A3 (4.2 μM)-, βA2 (4.5 μM)-, βA4 (4.5 μM)-, βB1 (4.0 μM)-, βB2 (4.4 μM)-, βB3 (4.3 μM)-, γA (0.13 μM)-, γB (0.13 μM)-, γC (0.13 μM)-, γD (0.13 μM)-, and γS (0.13 μM)-crystallin-GFP recombinant proteins in vitro was examined in pathological buffer, with or without 5% PEG8000. For (**a**,**b**), the incubation time was 10 min at 4 °C. *P < 0.05; **P < 0.01.

The crowding condition in the lens increases as age increases and this is a risk factor of crystallin protein aggregation^11^. Consistently, when PEG8000 was added to the solution to mimic the crowding environment in lens^12,13^, αA-, αB-, and βA2-crystallin-GFP proteins became aggregated, whereas other crystallin-GFP proteins remained soluble (Fig. 2b,c), suggesting that aggregation of αA-, αB-, and βA2-crystallin-GFP may be an early event of cataract. The concentration of crystallin proteins in human lenses is extremely high (about 450 mg/ml)^2^. When we increased the concentration of recombinant αA- or αB-crystallin-GFP proteins in the solution, the aggregates and the opacity were significantly increased (Figs. 2b, 3a, S1b,c).

**Figure 4:**
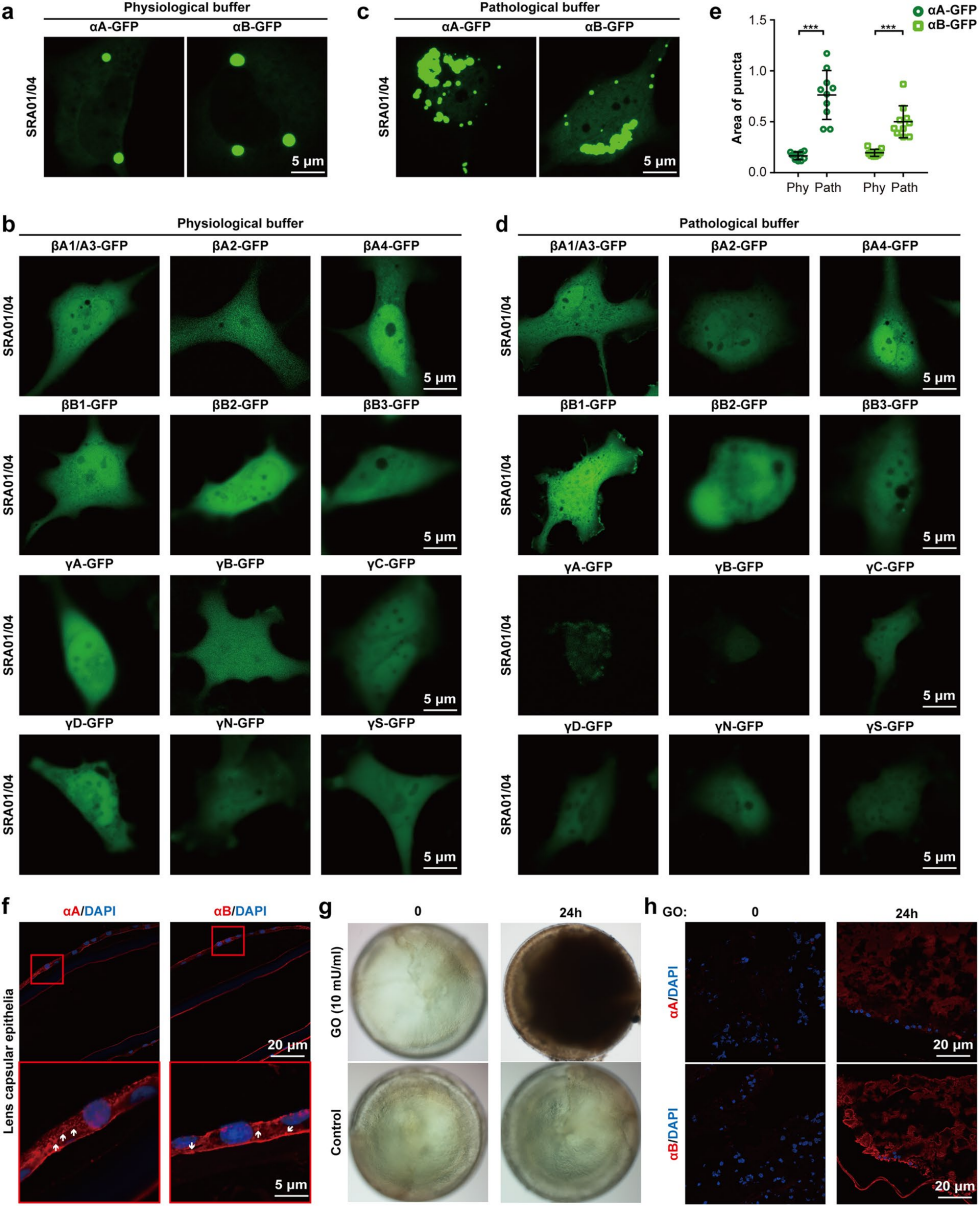
Live cell image of crystallin-GFP proteins in SRA01/04 cells in physiological or pathological buffer. (**a**,**b**) The crystallin-GFP protein-expressed SRA01/04 cells were incubated in physiological buffer. (**c,d**) The crystallin-GFP protein-expressed SRA01/04 cells were incubated in pathological buffer. For (**a**–**d**), cells were transfected with the indicated plasmid (1 μg) for 24 h, following by incubation with physiological buffer or pathological buffer for 30 min before imaging. (**e**) The fluorescence intensity of the α-crystallin-GFP puncta is presented as the area of the puncta. Data are the mean ± SD. n = 10 images for each group. *Phy* physiological buffer, *Path* pathological buffer. (**f**) Immunofluorescence of αA- and αB-crystallin in aging-related cataractous lens capsular epithelia. White arrows indicate αA- and αB-crystallin aggregates. (**g**) Treatment of mouse lenses with 10 mU/ml GO induced in vitro cataract. (**h**) Immunofluorescence of αA- and αB-crystallin in GO-treated mouse lenses. ***P < 0.001.

Disruption of lens epithelium due to increasing age or radiation-induced injury, the concentration of Na^+^ and Ca^2+^ will increase and K^+^ will decrease in the lens nucleus, which is one of the risk factors for cataract^14^. Consistently, the aggregation of recombinant αA-, αB-, and βA2-crystallin-GFP proteins largely increased in the solution containing high Na^+^, Ca^2+^, and low K^+^ (pathological buffer; Fig. 3a). Interestingly, several crystallin-GFP proteins that are soluble in physiological buffer formed aggregates in pathological buffer (Fig. 3b), indicating ion conditions are a strong inducer of crystallin aggregations. On the other hand, when we treated the crystallin-GFP-overexpressed lens epithelial cells with pathological buffer, only αA- and αB-crystallin-GFP proteins showed enhanced aggregation, while endogenous αA- and αB-crystallin remained soluble under a pathological environment (Figs. 4a–e, S1a). Most importantly, aggregated αA- and αB-crystallin proteins were also observed in aging-related cataractous lenses of patients (Fig. 4f).

### αB-crystallin-GFP is the major aggregated crystallin in oxidation- or diabetes-induced cataracts

**Figure 5:**
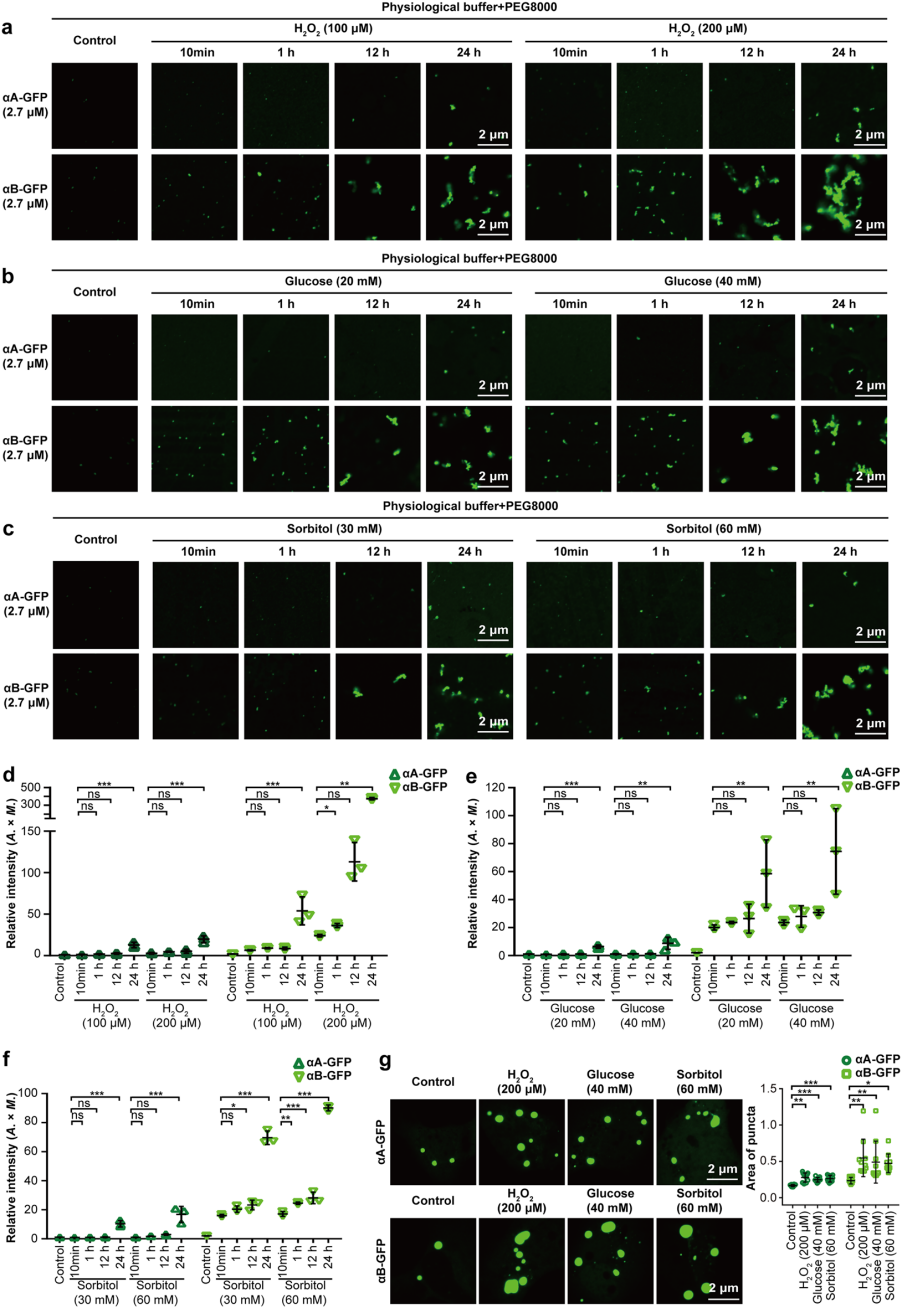
αB-crystallin-GFP is the major aggregated crystallin in oxidation- or diabetes-induced cataracts. (**a**–**c**) H_2_O_2_ (100 μM or 200 μM), high glucose (20 mM or 40 mM), and sorbitol (30 mM or 60 mM) slightly enhanced αB-crystallin-GFP (2.7 μM) but not αA-crystallin-GFP (2.7 μM) aggregation after incubation for 10 min at 4 °C in vitro. (**a–c**) αB-crystallin-GFP (2.7 μM) aggregation accumulated along with increases in the incubation times (1 h, 12 h, and 24 h), whereas αA-crystallin-GFP (2.7 μM) remained soluble. For (**a**–**c**), an in vitro assay was performed in physiological buffer with 5% PEG8000. (**d-f**) The fluorescence intensity is presented as the area × mean intensity (*A.* × *M.*). Data are the mean ± SD. n = 3 images for each group. (**g**) After transfection with αA- or αB-crystallin-GFP plasmids (1 μg) for 6 h, SRA01/04 cells were treated with H_2_O_2_ (200 μM), high glucose (40 mM), and sorbitol (60 mM) for 24 h before imaging. The fluorescence intensity of α-crystallin-GFP puncta is presented as the area of the puncta. Data are the mean ± SD. n = 10 images for each group. *P < 0.05; **P < 0.01; ***P < 0.001, *ns* no significance.

Oxidation is a major cause of age-related crystallin aggregation^15^. However, adding H_2_O_2_ to crystallin protein solutions only slightly promoted aggregation of αB-crystallin-GFP (Fig. 5a,d). Diabetes is a risk factor for cataract, which is mainly ascribed to hyperglycemia and the production of sorbitol^16,17^. Adding high glucose and sorbitol to crystallin protein solutions slightly promoted aggregation of αB-crystallin-GFP, but had no impact on αA-crystallin-GFP (Fig. 5b,c,e,f). The liquid-to-solid phase separation will increase along with time accumulation. Interestingly, as time increased, the aggregation of αB-crystallin-GFP was significantly enhanced, whereas αA-crystallin-GFP remained soluble (Fig. 5a–f), in the presence of H_2_O_2_, high glucose, or sorbitol. Consistently, adding H_2_O_2_, high glucose, or sorbitol to culture medium significantly enhanced the formation of αB-crystallin-GFP puncta in SRA01/04 cells, while endogenous αA- and αB-crystallin remained diffuse (Figs. 5g, S1a). Additionally, the aggregation of α-crystallin (mostly αB-crystallin) was also observed in glucose oxidase (GO)-induced cataracts of mouse lenses (Fig. 4g,h), indicating that the aggregation of α-crystallin was an early event of cataract.

## Discussion

Cataract, one of the leading causes of blindness worldwide, is the result of crystallin protein aggregation^7^. Early research showed that under pathological conditions, such as aging or radiation exposure, crystallin proteins could spatially separate into protein-rich regions and protein-poor regions, causing opacification and thus visual impairment^7,18^. In this study, we demonstrated that α-crystallin-GFP proteins formed aggregates via a phase separation mechanism.

Previous studies reported that α-crystallin, a member of the molecular chaperones^19^, prevents aberrant aggregation of damaged β- and γ-crystallin by interacting with the client protein using a variety of binding modes^20^. α-crystallin chaperone activity can be compromised by mutation or posttranslational modifications, leading to large-scale crystallin aggregation and cataract formation^21^. Surprisingly, in this study, we found that α-crystallin-GFP, without changes such as mutation or modification, could form condensates upon several risk factor stimulations. These observations suggested that α-crystallin may be the major aggregated crystallin in the early stage of cataract disease.

In the last century, cataract has been designated as a molecular condensation disease. Our results showed that although crystallin proteins remain soluble under normal conditions, aberrant crystallin condensates were largely induced under pathological conditions, such as aging and diabetes. Such aberrant condensates are also involved in neurodegenerative diseases^22–24^. For example, liquid droplets of FUS protein convert with time from a liquid to an aggregated state^22^. These findings indicated that aberrant phase transitions within liquid-like compartments are central for age-related cataracts. Previous studies have determined the phase separation of a protein-water mixture in cold cataract and selenite-induced cataract, which was associated with abnormal variation in temperature^18,25^. Annunziata et al*.* reported that γS-crystallin underwent liquid–liquid phase separation (LLPS), but this process needed an extremely low temperature (as low as − 8 °C)^26^. We did not observe the LLPS of γS-crystallin at room temperature, which was more like the situation peoples met. We thought that the low temperature might increase the multivalent interaction among γS-crystallin proteins, thereby accelerating the LLPS of γS-crystallin.

Previous reports suggested that protein oxidation can lead to formation of insoluble, light-scattering protein aggregates^27^. Another main risk factor of age-related cataract is diabetes^28^. For diabetes-related cataract, increased glucose and sorbitol concentrations in the lens are major initiators for crystallin aggregation^17^. Here, we found that although H_2_O_2_, glucose, or sorbitol only slightly promoted aggregation of αB-crystallin-GFP within a short time in vitro, as time increased, H_2_O_2_, glucose, or sorbitol significantly enhanced aggregation of αB-crystallin-GFP, whereas αA-crystallin-GFP remained soluble regardless the incubation time. These results, coupled with previous reports, illustrate that early oxidative and diabetic damage in crystallin proteins may be spontaneously reversed if oxidant and sorbitol are removed in time.

There are some shortages in this study. Firstly, all in vitro experiments were performed with crystallin-GFP protein. The big GFP tag may influence the properties of crystallin protein. Additionally, it is also reported that α-crystallin binding to lens membrane contributes to cataract formation^29^, and whether the aggregated α-crystallin bind to the lens membrane remains unclear. Finally, it needs to be further elucidated whether α-crystallin aggregation associates with other well-known causes of cataract, such as genetics, high myopia, smoking, medications, significant alcohol consumption, obesity, and hypertension^30^.

## Materials and methods

### Cell culture

Immortalized human lens epithelial cell line SRA01/04 was purchased from Shanghai Baifeng Biotech Co., Ltd. Another immortalized human lens epithelial cell line HLE-B3 was a gift form Prof. Ming-Xing Wu (State Key Laboratory of Ophthalmology, Zhongshan Ophthalmic Center, Sun Yat-Sen University). Both cell lines were mycoplasma-free and were authenticated using STR profiling by Guangzhou Cellcook Biotech Co., Ltd or American Type Culture Collection (ATCC). SRA01/04 and HLE-B3 were maintained at 37℃ in a 5% CO_2_ atmosphere and cultured in Dulbecco’s modified Eagle’s medium (DMEM, low glucose, Gibco, ThermoFisher Scientific, Waltham, MA, USA) supplemented with 10% fetal bovine serum (FBS, Gibco) and 100 units/mL penicillin–streptomycin (15,140,122, HyClone, South Logan, UT, USA). Cells were grown to 50–60% confluence before transfection with plasmids using Lipofectamine 2000 transfection reagents (ThermoFisher Scientific) according to the manufacturer’s instructions. For crystallin aggregation induction, cells were incubated with complete medium containing H_2_O_2_ (50 μM or 200 μM), glucose (10 mM or 40 mM), and sorbitol (15 mM or 60 mM) for 24 h, or treated with physiological buffer (20 mM NaCl, 10 μM CaCl_2_, 120 mM KCl, 20 mM Tris–HCl pH7.4, 0.6 mM glucose, 12 mM glutathione, 1 mM vitamin C, 5.9 mM inositol) or pathological buffer (150 mM NaCl, 30 mM CaCl_2_, 5 mM KCl, 20 mM Tris–HCl pH7.4, 0.6 mM glucose, 12 mM glutathione, 1 mM vitamin C, 5.9 mM inositol)^9,10,31^ for 30 min at 37 °C before subsequent analysis.

### Plasmid construction

The cDNA encoding crystallin was cloned into pGEX-6P-1 or pcDNA3.0 vectors. The cDNA fragments encoding our proteins of interest were generated with PCR using a NEBuilder HiFi DNA Assembly Cloning Kit (New England Biolabs, Beijing, China) and inserted in-frame before EGFP using the restriction enzyme sites including *BamH* I and *EcoR* I. Plasmid inserts were confirmed by Sanger sequencing (Tsingke, Guangzhou, China) and reading the full length of the insert. The human crystallin genes used in this study were as follows: α-crystallin (αA, αB), β-crystallin (βA1/A3, βA2, βA4, βB1, βB2, and βB3), and γ-crystallin (γA, γB, γC, γD, γN, and γS).

### Live cell imaging and fluorescence recovery after photobleaching (FRAP)

SRA01/04 or HLE-B3 cells were: (1) seeded on glass plates and transfected with crystallin-GFP plasmids for 24 h; (2) incubated with H_2_O_2_ (200 μM), glucose (40 mM), and sorbitol (60 mM) for 24 h, or (3) treated with the physiological buffer for 30 min before imaging; then, the physiological buffer was discarded and cells were further incubated with the pathological buffer for another 30 min before imaging. Confocal images were taken with a Zeiss LSM880 confocal microscope with a 488 nm laser using a 60X oil immersion lens. Images were processed with ZEN software (Blue edition, 3.1). Fluorescence intensity was measured with Image J. For FRAP experiments, the green puncta were bleached with 100% laser power (488 nm), and time-lapse images were captured every 1 s. Images were further processed using ZEN3.1, and the fluorescence intensity was normalized to the prebleaching time points. GraphPad Prism was used to plot and analyze the FRAP results.

### Protein purification

Crystallin-GFP recombinant proteins were expressed in *Escherichia coli* BL21(DE3). *E*. coli cells were grown to OD600 of 0.6 at 37 °C and induced with 0.5 mM IPTG (R0393, Invitrogen, Waltham, MA, USA) at 16 °C for 16 h. Cells were harvested by centrifugation at 4000*g* for 10 min at 4 °C, resuspended in 1 × PBS (supplemented with 1 mM PMSF), and then lysed by sonication. Lysates were centrifuged twice at 10,000*g* for 20 min at 4 °C. The supernatant was subjected to the purification of crystallin-GFP proteins using a GST-tag protein purification kit (P2262, Beyotime, Shanghai, China) according to the manufacturer’s protocol. Consequently, the eluted proteins were confirmed by SDS-PAGE and stored at − 80 °C.

### In vitro aggregation

For in vitro aggregation experiments, purified crystallin-GFP proteins were diluted to the indicated concentrations in physiological buffer or pathological buffer with or without 5% PEG8000, and then incubated at 4 °C for 10 min. In vitro aggregation experiments were also performed to investigate crystallin protein aggregation in response to H_2_O_2_ (100 μM, 200 μM), glucose (20 mM, 40 mM), and sorbitol (30 mM, 60 mM). Finally, 10 μL of each mixture were placed on a glass slide or a 384 well glass bottom plate for imaging with a Zeiss LSM880 confocal microscope. All images were processed with ZEN3.1. Fluorescence intensity was measured with Image J. For opacity analysis, we performed 200 μL reaction mixtures and measured the apparent absorbance at 400 nm to detect the opacity of α-crystallin aggregates in vitro.

### Assay of chaperone activity

Chaperone activity of purified recombinant αA- and αB-crystallin-GFP proteins was measured at 25 °C using an insulin B-chain aggregation assay as described previously^32^. Briefly, insulin (0.35 mg/mL, in 50 mM PBS pH 7.2) was reduced with 20 mM DTT. Aggregation was monitored in the presence of αA-crystallin (1.05 mg/mL), αB-crystallin (0.35 mg/mL), or both αA- and αB-crystallin in a 96-well plate by measuring the apparent absorbance at 400 nm after the indicated incubating time (0, 15, 30, 45, or 60 min).

### Collection of human lens capsular epithelial samples

Collection of human capsular epithelia from cataract lenses was approved by the Institutional Research Ethics Committee of the Sixth Affiliated Hospital of Sun Yat-sen University. Informed consent was obtained from each of the cataract patients. All procedures followed the ethical principles of the World Medial Association (WMA) Declaration of Helsinki. The lens capsules from 10 cataract patients were collected at surgery by the physicians in Guangdong Provincial People’s Hospital. The clinical classifications of cataract patients are summarized in Table 1. Cataract grade was evaluated according to the Lens Opacities Classification System III^33^.

**Table 1:** Cataract patients’ information.

Number	Gender	Age	Classification
1	Male	55	C2N2
2	Female	58	C2N2
3	Female	76	C2N2
4	Male	61	C2N2P2
5	Male	68	C2N3
6	Male	71	C3N2
7	Male	67	C2N3P1
8	Male	55	C2N3P3
9	Male	44	C3N4P2
10	Female	63	C3N5P2

*C* cortex, *N* nuclei, *P* posterior capsule.

### Animals

Animal experiments were approved by the Institutional Animal Care and Use Committee of the Sixth Affiliated Hospital of Sun Yat-sen University. The experimental procedures with animals complied with ARRIVE guidelines and were performed in accordance with the U.K. Animals (Scientific Procedures) Act, 1986. Four-week-old male C57BL/6 J mice were purchased from Gempharmatech-GD (Guangdong, China). The eyeballs of the mice were removed and the lenses were carefully dissected after sacrifice with CO_2_ inhalation. Dissected lenses were placed in a 10-cm dish containing 20 ml Medium 199 (M4530, Sigma-Aldrich), and incubated at 37 °C in a 5% CO_2_ atmosphere for 12 h. Then, three transparent lenses were transferred into a 6-cm dish and incubated with 8 ml Medium 199 containing 10 mU/ml glucose oxidase (GO, G7141, Sigma-Aldrich)^34^, which continuously generated oxidative stress and induced crystallin aggregation. The morphological and crystallin protein changes of the lenses were analyzed at 0 and 24 h after GO treatment.

### Immunofluorescence analysis

SRA01/04 or HLE-B3 cells seeded on glass coverslips were fixed with 4% paraformaldehyde for 15 min. Frozen human lens capsular epithelia from cataract lenses and the entire GO-treated mouse lenses were fixed with cold acetonum for 10 min. After fixation, cells or samples were incubated with blocking buffer (5% goat serum, 0.3% Triton X-100 in 1 × PBS) for 1 h and primary antibodies containing blocking buffer for 2 h at room temperature. After three washes in 1 × PBS, cells or samples were incubated with secondary antibodies tagged with Alexa Fluor 488, 555, or 647 (4408S, 4413S, or 4414S, Cell Signalling Technology) for 1 h at room temperature in the dark, following by DAPI staining (D9542, Sigma-Aldrich) for 5 min. Images were acquired using a Zeiss LSM880 confocal microscope and processed with ZEN3.1. The primary antibodies used in immunofluorescence analysis included: CRYAA (A5725, ABclonal), CRYAB (A9633, ABclonal), Vimentin (A19607, ABclonal), and DYNC1H1 (12345-1-AP, Proteintech).

### Statistical analysis

All data were expressed as mean ± standard deviation (SD) of independent experiments performed in triplicate. Statistical analyses were performed with SPSS 20.0 software (SPSS, Inc., Chicago, IL). Unpaired *t* test was used to assess the difference between two groups and one-way analysis of variance were used when more than two groups were compared. The *p*-value < 0.05 was considered statistically significant. *, **, and *** represent *p* < 0.05, *p* < 0.01, and *p* < 0.001, respectively.

## Supplementary Information

Supplementary Figures.

## Data Availability

The raw/processed data required to reproduce these findings can be obtained from the corresponding author upon reasonable request.
