# Supplementary material for: Phase separation of α-crystallin-GFP protein and its implication in cataract disease
Source: Sci Rep. 2023 Mar 24;13:4832. doi: 10.1038/s41598-023-31845-9 (PMC10039050; doi:10.1038/s41598-023-31845-9)

Supplementary Figure and Legend

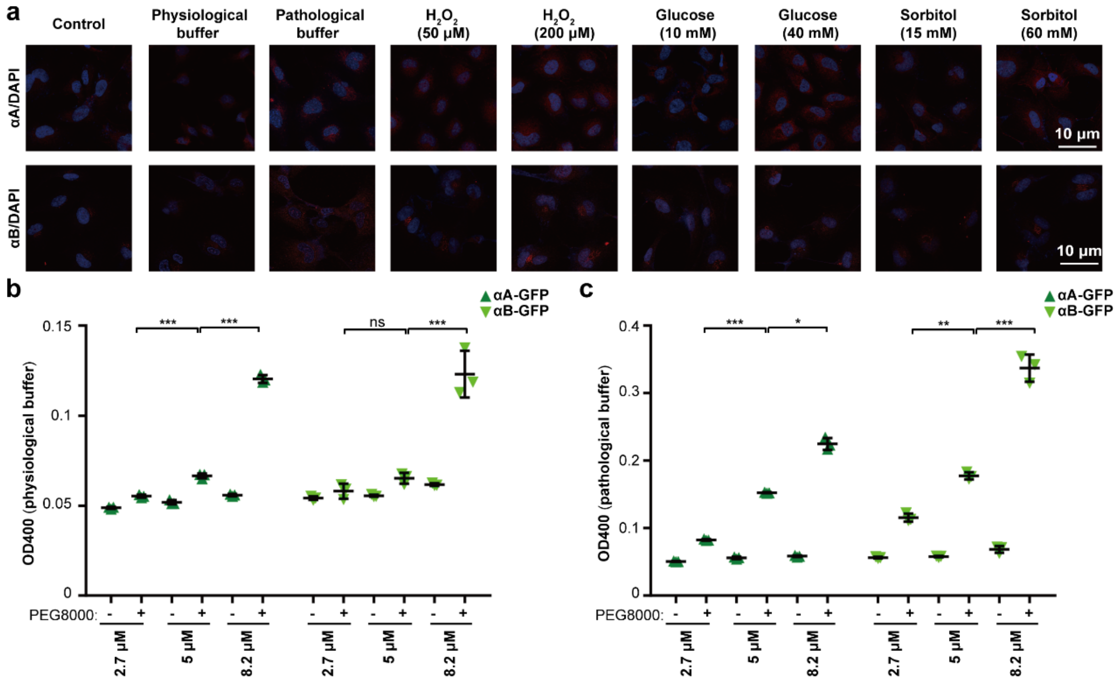

Figure. S1 Endogenous αA- and αB-crystallin under physiological and pathological environments, or H_2O_2, high glucose, and sorbitol treatment. (a) Cells were incubated with physiological buffer and pathological buffer for 30 min, or H_2O_2 (50 μM or 200 μM), high glucose (10 mM or 40 mM), and sorbitol (15 mM or 60 mM) for 24 h before an immunofluorescence assay. (b-c) Analysis of opacity when α-crystallin-GFP form the aggregates.

Original images: Figure 2a

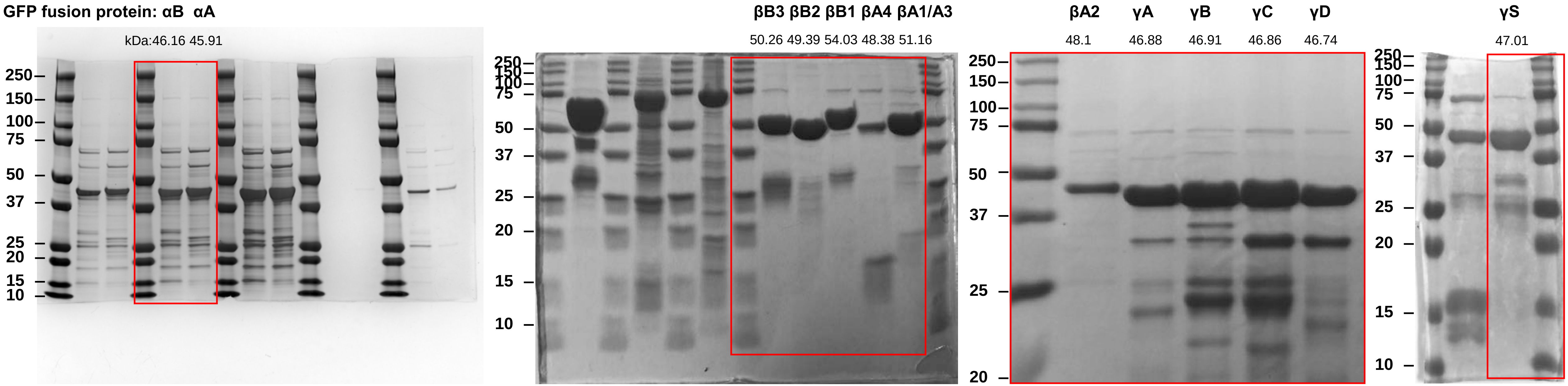

Supplement: Supplementary file 1 — Supplementary Figures. [file 41598_2023_31845_MOESM1_ESM.pdf]
